# Agency and Performance of Reach-to-Grasp With Modified Control of a Virtual Hand: Implications for Rehabilitation

**DOI:** 10.3389/fnhum.2020.00126

**Published:** 2020-04-23

**Authors:** Raviraj Nataraj, Sean Sanford, Aniket Shah, Mingxiao Liu

**Affiliations:** ^1^Movement Control Rehabilitation (MOCORE) Laboratory, Stevens Institute of Technology, Hoboken, NJ, United States; ^2^Department of Biomedical Engineering, Stevens Institute of Technology, Hoboken, NJ, United States

**Keywords:** cognitive agency, reach to grasp, movement rehabilitation, virtual reality, visual feedback

## Abstract

This study investigated how modified control of a virtual hand executing reach-to-grasp affects functional performance and agency (perception of control). The objective of this work was to demonstrate positive relationships between reaching performance and grasping agency and motivate greater consideration of agency in movement rehabilitation. We hypothesized that agency and performance have positive correlation across varying control modes of the virtual hand. In this study, each participant controlled motion of a virtual hand through motion of his or her own hand. Control of the virtual hand was modified according to a specific control mode. Each mode involved the virtual hand moving at a modified speed, having noise, or including a level of automation. These specific modes represent potential control features to adapt for a rehabilitation device such as a prosthetic arm and hand. In this study, significant changes in agency and performance were observed across the control modes. Overall, a significant positive relationship (*p* < 0.001) was observed between the primary performance metric of reach (tracking a minimum path length trajectory) and an implicit measurement of agency (intentional binding). Intentional binding was assessed through participant perceptions of time-intervals between grasp contact and a sound event. Other notable findings include improved movement efficiency (increased smoothness, reduced acceleration) during expression of higher agency and shift toward greater implicit versus explicit agency with higher control speed. Positively relating performance and agency incentivizes control adaptation of powered movement devices, such as prostheses or exoskeletons, to maximize both user engagement and functional performance. Agency-based approaches may foster user-device integration at a cognitive level and facilitate greater clinical retention of the device. Future work should identify robust and automated methods to adapt device control for increased agency. Objectives include how virtual reality (VR) may identify optimal control of real-world devices and assessing real-time agency from neurophysiological signals.

## Introduction

Sense of agency during movement intuitively leads to better physical function, but it is not a primary rehabilitation target compared to increased strength or practiced skill (Shepherd, 2001; Yang et al., 2006; Timmermans et al., 2009). Powered devices such as exoskeletons (Rosen et al., 2001; Heo et al., 2012) and prosthetics (Childress, 1973; Li et al., 2010), can inject the mechanical energy to physically assist the user. However, functional performance depends on how well the person can control the device toward intended actions. The ability to control these assistive devices primarily depends on a robust command interface from which the user can reliably trigger device actions. The command interface can infer user intention from mechanical triggers such as switches (Bhadra et al., 2002; Peckham and Knutson, 2005). More “natural” interfaces involve command detection from computational processing of recorded physiological signals such as muscle electromyography (EMG) (Boostani and Moradi, 2003) or brain electroencephalography (EEG) (Wolpaw and McFarland, 2004). Despite the interface, functional control is generated from the user’s ability to cognitively integrate their intention with observed device actions toward desired performance outcomes. This study investigated how modifying control of a virtual hand executing reach-to-grasp contributed to performance of functional reach and sense of grasp agency. It was hypothesized that control modes inducing higher agency would also demonstrate greater performance. To verify this relationship as broadly applicable, we investigate control modes that are diverse (changes in speed, presence of noise, addition of automation). Such positive associations should motivate greater consideration of agency in movement rehabilitation.

Sense of agency is defined as the perception of control over actions and related sensory consequences (Moore and Obhi, 2012). Since sensorimotor control of functional movements involves sequences of motor actions continually modulated by sensory feedback (Todorov, 2004), measuring agency by action-consequence events may be especially pertinent and effective in methods to rehabilitate movement. Significant previous work has demonstrated conditions under which sense of agency is generated and modulated (Moore, 2016; Haggard, 2017, 2019). These conditions include voluntary versus involuntary movements (Haggard et al., 2002), matching actual and expected consequences (Frith et al., 2000; Blakemore et al., 2002), and the effects of external cues (Moore et al., 2009). Thus, experimental conditions may be constructed to provide cues that boost agency, but it is unclear if greater agency is related to better movement performance and which conditions may precipitate both. If clear links between agency and movement performance were established, methods to adapt device control for better cognitive engagement and ability with a device may be better pursued. Greater perception of control would naturally engage the user, and user ability is inherently reflected through greater performance. Engagement and ability are vital factors for clinical retention of device-based rehabilitation. Such approaches are especially beneficial for developing sensorimotor prostheses (Marasco et al., 2018) and powered exoskeletons (Farris et al., 2013) that restore function after neurological trauma. Individuals with brain injury, spinal cord injury (SCI), or amputation may undergo intensive therapy to improve both physical and cognitive skills in re-learning functional movements with devices.

A major advancement in rehabilitation device technology would be the creation of methods that not only optimize user-device mechanics but also cognitive engagement of the user. Systematically identifying user agency and adapting device control accordingly may produce better performing, cognition-driven rehabilitation devices. Ultimately, clinical retention of rehabilitation devices is predicated on user perception of utility (Phillips and Zhao, 1993; Hughes et al., 2014). Methods that leverage perception metrics, such as agency, can also facilitate more usage of rehabilitation devices. Devices for rehabilitation are those that improve movement function for persons with neuromuscular dysfunction. We classify devices either providing powered movement assistance or training for independent function through robotic and computer interfaces as rehabilitation devices. In both cases, greater cognitive engagement and involvement due to user agency in controlling the device should facilitate better, and more natural, performance.

Intentional binding is an established implicit measure for agency. It indicates how coupled one perceives an intended action to an expected sensory consequence (Haggard et al., 2002; Moore and Obhi, 2012). Intentional binding refers to the perceived compression in time between a movement and its consequences during voluntary control (Haggard et al., 2002). The classical construct for intentional binding involved action of a key press to trigger the delayed onset of a sound tone. Participants would judge the time duration between key press and tone. A perceptual shift toward compression of time was shown when the key press was voluntary versus an involuntary twitch induced by transcranial magnetic stimulation. This binding effect is considered implicit since it is specific to voluntary action while passively induced actions can produce a reversal of this effect (Moore et al., 2012). Intentional binding has been used to show the influence of sensorimotor processes on agency through internal prediction and external action outcomes (Haggard et al., 2002; Moore and Haggard, 2008; Moore and Obhi, 2012; Frith and Haggard, 2018). Physical rehabilitation methods could be well served to monitor agency during the recovery and reformulation of sensorimotor pathways after neurotrauma. Intentional binding metrics for agency have already been used for human computer interaction to show the sensitivity of implicit agency to particular input modalities (Coyle et al., 2012; Limerick et al., 2014). Furthermore, it has been shown that brain machine interfaces (BMIs) can generate experiences of explicit agency in users similar to bodily movements (Evans et al., 2015). Explicit agency requires subjects to provide higher-order, conscious assessments of perception of control for given conditions (Moore et al., 2012). Given the sensitivity of both implicit and explicit agency to external cues, a variety of sensory feedback paradigms may be employed to train user-device integration centered on agency. As such, the effects of varying device control on both implicit and explicit agency should be examined.

Virtual reality (VR) is an attractive platform to develop customized methods for user-device integration and agency-based rehabilitation. For the user, VR is proven to enhance cognitive engagement in performing repetitive physical therapy movements (Sveistrup, 2004; Saleh et al., 2017). VR is readily programmable (Todorov et al., 2012) to customize visual projections of user actions and their consequences in functional task performance. Visual feedback from VR can modulate for both sense of agency (Moore and Fletcher, 2012) and control of functional movements like reaching (Desmurget and Grafton, 2000; Saunders and Knill, 2003; Nataraj et al., 2014b) and grasping (Winges et al., 2003; Nataraj et al., 2014a). Reach-to-grasp is a fundamental human action and is commonly targeted for rehabilitation following neuromuscular dysfunction (Lin et al., 2007; Loureiro and Harwin, 2007) and can be assisted with powered devices triggered by user command actions (Popovic, 2003; Kotecha et al., 2014). With neurotrauma such as SCI, visual capabilities are still largely intact and can be leveraged further in VR to partially compensate loss of other senses (Ghez et al., 1995) such as touch and proprioception. For rehabilitation devices, such as prostheses and exoskeletons, VR platforms can be flexibly constructed to train complex interfaces involving direct physiological access (Kuiken et al., 2009; Marasco et al., 2018) or powered actuation of limbs (Hartigan et al., 2015). VR could be employed to match user intentions to optimal parameters for controlling a device using visual projections of device actions following user commands. Control parameters include feedback gains to maximize performance and minimize effort (Nataraj and van den Bogert, 2017) and to achieve desired movement features such as smoothness (Hogan and Sternad, 2009). Ultimately, VR platforms may be utilized to efficiently identify control parameters of rehabilitation devices that optimize not only functional mechanics but also user agency prior to eventual translation to real-world systems (Caldwell et al., 1995, 1998; Bar-Cohen, 2003; Perry et al., 2007).

In this study, a VR environment was utilized to couple reach and grasp “actions” to programmed sensory “consequences” (visual and sound events). Participants triggered movement control of the virtual hand through movement of their own hand. The visually observed movement of the virtual hand depended on the specific control mode. The control mode defined at what fixed speed the virtual hand would move proportional to the real hand and if virtual movement included noise or assisted automation. We investigated how changes in user control of a virtual hand prosthesis (Johannes et al., 2011) during reach-to-grasp may generate effects across both sense of agency and functional task performance. Visual cues informed the participant about initiating and pacing the reach, where to grasp, and when grasp action was successfully completed. The primary performance metric was reducing position error of the participant’s hand to a minimal path-length trajectory at a fixed velocity. As with previous intentional binding studies (Moore and Obhi, 2012), a sound cue (beep) was used as the consequence to an intended action (grasp). Participants provided verbal estimates of lapsed time intervals between action and consequence to infer agency implicitly via intentional binding across the various control modes. The control modes of the virtual hand were consistent with parameters commonly adapted for a movement rehabilitation device, and included: setpoints for speed (Blaya and Herr, 2004; Wege et al., 2005), noise mitigation (Taylor et al., 2002; Agostini and Knaflitz, 2012), and a level of automated assistance (Ronsse et al., 2010, 2011). Speed, noise, and automation are fundamental control parameters that a device engineer can *ad hoc* tune based on stated user preferences or anecdotal observation of performance (Terenzi, 1998). Alternatively, these parameters can also be determined through optimization of mechanical performance (e.g., effort, tracking) for a model system (Davoodi et al., 2007; Nataraj and van den Bogert, 2017). Neither approach systematically adapts the device according to user agency. The major implication of this study is how a subjective metric of perception in control of a virtual device (hand) can be related to objective performance (reaching) with that device. In this study, the control modes were enacted as deviations from an optimal (“Baseline”) mode, at which the virtual hand moved to match the actual hand movements and agency is expected to be highest.

Unlike previous studies that identified agency for movement initiation (Haggard et al., 2002), this study investigated how agency of grasp execution was modulated by the control mode of the preceding reach. In this way, it was inferred how control *during* reaching may facilitate or inhibit agency of the terminating action of grasp and performance of the reach itself. Previous studies have shown the direct link of agency between continuous movements and terminal events (Wen et al., 2015; Oishi et al., 2018). In this study, we prioritized and considered implicit agency by time-interval estimation as a less biased (more sub-conscious) perceptive measure. With time-interval estimation, a quantifiable measure was provided each trial that was not readily linked to a conscious preference to a control mode. The main hypotheses of this VR reach-to-grasp study were: (1) implicit grasp agency and reaching performance are positively related across a broad class of control modes typically considered for rehabilitation devices, (2) significant differences in both implicit agency and performance are observable between these control modes. While our primary hypotheses considered implicit agency, we additionally examined explicit perception of each control mode with Likert-scale survey responses. The purpose of the survey responses was to observe how implicit and explicit agency may be related through the presented control modes of this study. Another important implication of translating agency to more effective rehabilitation device control is greater performance efficiency. Thus, the secondary hypotheses of this study were: (1) there are significant shifts between implicit and explicit agency across control modes, (2) agency is positively related to performance efficiency, and (3) significant differences in efficiency exist between control modes.

## Materials and Methods

**FIGURE 1:**
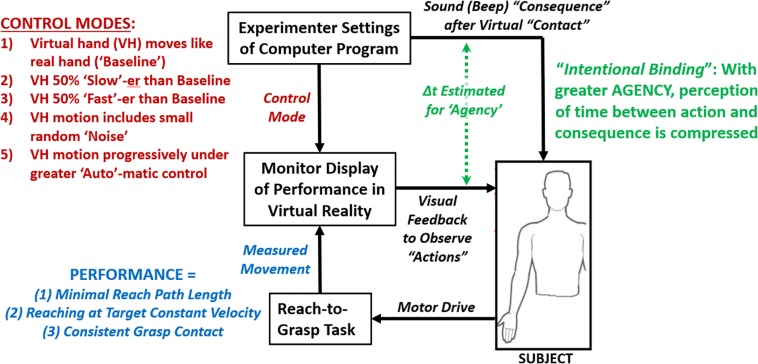
Flow diagram of experiment of participant performing reach-to-grasp task under varying control modes of displayed virtual hand while assessing performance and agency.

In this experimental protocol, participants controlled a virtual hand to perform reach-to-grasp through movements of their own hand (Figure 1). The observed movement of the virtual hand were initially based on those of the real hand (“Baseline” case) but modified depending on the other control modes tested. The modifications from Baseline involved fixed changes in speed, addition of noise, or inclusion of automation. Participants were asked to maximize performance (primarily moving own hand to minimize reaching path length at a target velocity) and provide verbal estimates of perceived time-intervals between grasp action and a sound consequence for implicit assessment of agency.

### Participants

A total of 16 able-bodied volunteers (12 male, 4 female, 20.9 ± 3.2 years) were recruited to participate in this study. A power analysis for one-way ANOVA at 95% suggested that eight-participant samples would show significant differences (α = 0.05) in implicit agency and reaching performance. In this power analysis, performance was for minimizing path length (see “Data and statistical analysis”) across the tested control modes during a pilot study (Shah et al., 2018). Only right-handed participants were tested for right-hand reach-to-grasp to avoid considering effects of hand dominance. All participants had normal or corrected-to-normal vision and did not previously report nor demonstrate a history of disease, injury or complications involving cognition or upper extremity function. All participants signed an informed consent form approved by the Stevens Institutional Review Board.

### Equipment (Hardware and Software)

A marker-based motion capture system was used to track 3-D hand motions and correspondingly control a virtual model of a prosthetic hand [MPL, *Modular Prosthetic Limb* (Johannes et al., 2011)]. The hand was viewed in a VR environment with advanced contact mechanics [*Multi-Joint Dynamics with Contact*, *MuJoCo*, Roboti LLC, Seattle, Washington, United States (Todorov et al., 2012)]. The motion capture system included nine infra-red cameras (*Prime 17W* by Optitrack, NaturalPoint Inc., Corvallis, OR, United States) to track 3-D position and orientation of three retroreflective marker clusters. The first cluster included three markers (9 mm diameter) that were Velcro-affixed in a non-colinear arrangement on a worn glove at the dorsal side of the hand (midpoint of third metacarpal). This “hand” cluster served as a reference coordinate system mapping real-time changes in position and orientation to the virtual hand. Similarly, two additional clusters with smaller markers (4 mm diameter) were placed on the nails of the index finger and thumb. These nail clusters were affixed to 3-D printed platforms that attached to the nails using double-side adhesive tape. Coordinate systems represented by these nail clusters drove position and orientation of the distal segments of the respective digits. Joint angle changes across the digits were based on real-time inverse kinematics solutions sufficiently satisfying the position and orientation constraints of all three clusters. Position constraints for the nail clusters were relative to the hand cluster and scaled for each participant hand size to match the virtual hand size. Only the thumb and index finger were tracked and animated on the virtual hand as the functional task was reach to precision grasp (Nataraj et al., 2014a), requiring focus onto smaller objects. Real-time streaming of marker data to manipulate the VR environment was done using the motion capture software (*Motive* by Optitrack) and API code written in MATLAB (Mathworks Inc., Natick, MA, United States) running on a Dell Workstation. All data was processed at 120 Hz.

### Protocol

#### Participant Preparation

**FIGURE 2:**
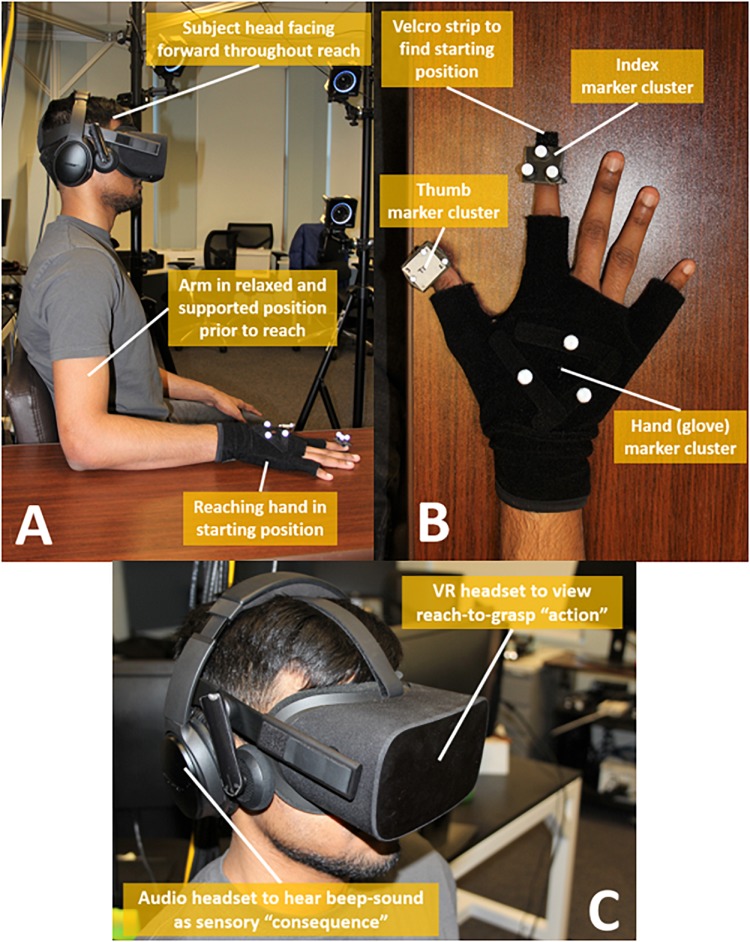
Experimental set-up elements. **(A)** Participant positions body and arm at start of each trial, **(B)** Hand shown with motion capture marker clusters, **(C)** Participant head mounted with VR headset and audio headset.

Upon arriving to the laboratory, participants were re-informed about protocol and their right-hand size was measured. Hand size was measured as the maximum spread distance from tip of thumb to tip of index finger. The average hand size was 15.2 ± 0.95 cm. For each participant, hand size was used to spatially calibrate motions of the index finger and thumb clusters relative to the hand cluster of the real hand to those of the virtual hand. Each participant was seated with chair height adjusted so that the reaching arm would be table-supported to initially have: the elbow at a right angle, shoulders comfortably level, and upper-arm at the participant’s side (Figure 2A). Each participant then wore a glove (Figure 2B) with hand marker cluster attached. A marker cluster was then added to each of the index finger and thumb nails. The participant then had placed over their head and eyes an Oculus^®^ *Rift* headset (Facebook Technologies, LLC) displaying a custom virtual environment (*MuJoCo*) as seen in Figure 2C. The participant then had placed over their ears a noise canceling headset (Bose^®^ *QuietComfort 35*) to minimize audible distractions and primarily only hear an occasional beep tone (sound consequence) as part of the experimental task.

#### Virtual Reality Calibration Procedures

The Oculus display filled the participant’s entire field of view with the virtual environment. Participants were able to find an initial starting position for their real hand based on tactile sensation of a Velcro strip on the support table. The view within the virtual environment was initially calibrated such that the hand marker cluster position of the real hand was coincident with the same landmark position of the virtual hand. In front of the participant’s virtual view was a sphere (7 cm diameter) that served as the target the participant reached toward and grasped each trial. The virtual sphere was located 20 cm above and 25 cm anterior to the initial hand cluster position. Two tracks for speed pacers were also within view. One pacer moved forward and the other vertically to inform the participant about the target hand velocity in each dimension. The tracks were semi-transparent to subtly cue the participant about speed without distracting visual focus from the virtual hand. The pacer speeds were set to traverse each dimension in 4 s.

#### Virtual Reality Task

**FIGURE 3:**
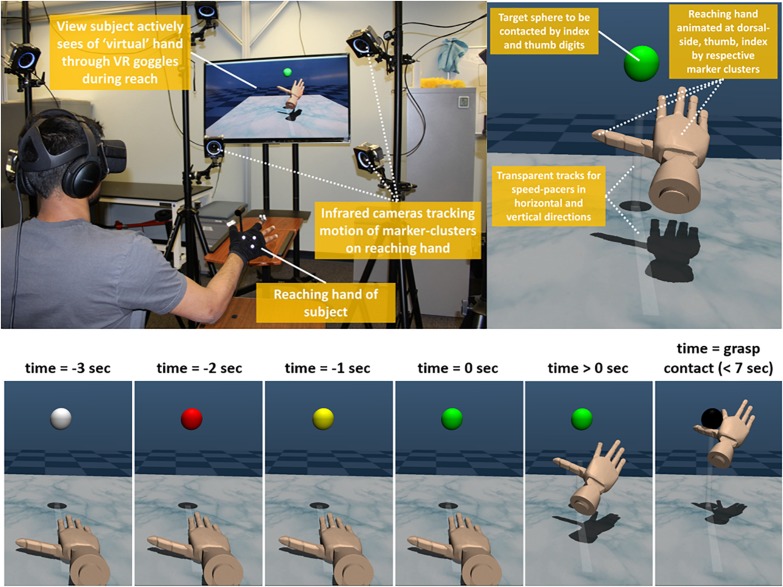
Virtual reality environment. (*Top Left)* Participant actively reaches and concurrently views virtual hand, (*Top Right)* Close-up of virtual hand reaching to target sphere against transparent speed pacer tracks, *Bottom)* Sphere color changes with hand transitions across trial time *t* = –3 to +7 s (10 s total). Countdown occurs from *t* = –3 to 0 s. After countdown, hand should be in “motion” during time sphere is green.

Each trial, the participant was cued by countdown to begin performing reach-to-grasp (Figure 3). The countdown for a trial was represented by color transitions of the target sphere as follows: red at trial time (*t*) = −2 s, to yellow at *t* = −1 s, and to green at *t* = 0 sec, at which time the speed pacers, moving at constant velocity, began to move and the participant should initiate hand movement. The pacers ceased movement after *t* = 4 s or earlier when the participant made premature grasp contact. Participants were told to maximize reach-to-grasp performance across three criteria: (1) minimize reaching path length, (2) match hand reaching velocity to speed pacers and complete reach-to-grasp in precisely 4 s, and (3) grasp the target sphere with thumb and index finger at consistent locations. Participants were told that reaching performance was primarily evaluated in this study but to self-consider all three performance criteria to promote task consistency. Each trial lasted up to 10 s as the participant had 7 s to complete reach-to-grasp with the goal to complete in precisely 4 s. Although natural reach-to-grasp is executed nominally at 1 s (van Vliet and Sheridan, 2007), reaching time with a neural controlled robotic device can be notably slower (∼6 s) (Hochberg et al., 2012). In this study, ecological validity for reach performance and grasp agency was intended more for device control.

When the virtual hand grasped the target sphere with both the index and thumb digits, the sphere instantly changed color from green to black and the virtual environment froze in place. This color-change event cued the participant that grasp action was successfully completed. A short-duration (∼100 ms), moderate-pitch beep was sounded to the participant’s headset at a variable time-interval following grasp action. The participant was asked to verbally estimate the time-interval to the best of their abilities after each beep. The participant was previously instructed that the interval for each trial was anywhere from 100 to 1000 ms in denominations of 100 ms. The actual intervals were always 100, 300, 500, 700, or 900 ms. For each block of trials to test a specific control mode, the number of trials presented at a given time-interval was based on a Gaussian distribution centered at 500 ms. This approach in presenting time-intervals was modified from previous intentional binding experiments that assessed agency with a uniform distribution of intervals at 300, 500, and 700 ms (Caspar et al., 2015). Pilot data revealed that these modifications facilitated a distribution of estimates necessary to infer differences in agency across several control modes (6 in all, see next section). Greater underestimation of time-intervals indicated greater compression (shortening) of the perceived time-interval and implicitly demonstrated greater agency (Haggard et al., 2002). As with other intentional binding experiments, our implicit measure of agency served as a more sub-conscious perception of control.

#### Varying Control Modes

Each participant performed the reach-to-grasp task under six different control modes. As previously described, the control modes examined in this study considered modifications in speed, addition of mild noise, and automation. The participant was aware of each control mode being tested through visual feedback of the virtual hand in reference to their own moving hand. The test cases of control modes were as follows:

(1) Grasp-Only –The virtual hand was initially placed near the target sphere whereby no reach was required, and only grasp action was needed to complete the trial. This test case served as a control for all subsequent control modes to observe how a preceding movement phase (reach) may affect agency of the terminal action (grasp). As previously described, other studies have investigated agency in relation to the intent to move with a simple key press (Haggard et al., 2002). Our study examined a more complex functional task with two components (reach and grasp) whereupon movement was already initiated prior to grasp. This case with grasp-only was analogous to initiation of key press. All other control modes tested included reach and grasp.
(2) Baseline –The virtual hand moved in equal proportion to the real hand in all three dimensions. This control mode was tested once at the beginning of the session (after grasp-only) and repeated at the end. The first test block was used for comparison to other cases. The second block was done to compare agency and performance to the first block and verify possible changes due to fatigue or learning across the session.
(3) Slow –The virtual hand moved in all three dimensions at a speed that was 50% slower than the real hand. The virtual hand appeared “sluggish,” and the participant needed to move the real hand 50% faster and further as compensation to control the virtual hand and complete reach-to-grasp as intended.
(4) Fast –The virtual hand moved in all three dimensions at a speed that was 50% faster than the real hand. The virtual hand appeared “hyperactive,” and the participant moved the real hand 50% slower and shorter to compensate and control the virtual hand as intended.
(5) Noise –The virtual hand was infected by mild to moderate noise. A small random value was added in each of the three dimensions for the position of the real hand. The random value was ±*X*, where *X = 10 × displacement from previous time-step*. Given the sampling frequency of 120 Hz, the noise amplitude was proportional to hand velocity as ±1 cm per 12 cm/sec. This noise-level produced light visual tremor to the moving hand that was clearly noticeable but not overtly distracting or challenging to complete the reach-to-grasp task.
(6) Auto –The virtual hand was progressively (linear with time) under automatic control. At the start-time of reach (*t*_*reach*_ = 0), the participant controlled the virtual hand just as in “Baseline.” Over the designated 4-s reach duration, the position of the virtual hand (*pos*_*VR–hand*_) was a weighted average of the participant’s real hand position (*pos*_*subj*_) and a pre-defined optimal position (*pos*_*opt*_) corresponding to the minimal path trajectory. The virtual hand position was given as: $pos_{VR-hand}=\left(1-\frac{t_{reach}}{4}\right)\times pos_{real}+\left(\frac{t_{reach}}{4}\right)\times pos_{opt}$. At *t*_*reach*_ = 4 s, the virtual hand was guaranteed to be very near the sphere, but the participant must still volitionally perform grasp to complete the trial. This automated case was akin to user initiation of movement to trigger device assistance and auto-complete the movement (Lucas et al., 2004).

#### Experimental Testing Blocks

Participants would perform a block of 20 consecutive trials for each of the six control modes. The first three trials of every block were “practice” with the time-interval between grasp contact and the beep fixed at 1 s. The participant was aware these practice trials served to gain mild familiarity with the control mode and to re-calibrate their internal reference of a 1 s time-interval. The remaining 17 test trials were used for agency and performance assessment with time-intervals to be estimated ranging from 100 to 1000 ms as previously described. After each trial, the VR hand was reset to the initial position prior to the 3-s countdown to initiate movement for the second trial. Each participant was given up to 5 min between blocks to rest and complete a survey to rate their experience for that block.

#### Surveys

After each block, the participant was presented with a 1-statement survey to express their subjective perception of the control mode presented. Participants were asked to rate, on a 5-point Likert scale (−2 = strongly disagree, +2 = strongly agree), to what extent they agreed that the visualized hand motions reflected their intentions. The specific statement read “the visualized hand motions reflected your intentions.” The survey responses served as an explicit, or conscious, measure of agency (Moore et al., 2012; Dewey and Knoblich, 2014) for each control mode. The single survey was presented at the end of each block to ensure subjects accommodated to a control mode prior to making a conscious subjective assessment.

### Data and Statistical Analysis

The primary performance metric evaluated across control modes was the inverse of path length error to a minimal path length trajectory occurring at constant velocity over 4 s. The total 3D minimal pathlength was 0.32 m, and for completion in 4 s, the target constant velocity is 0.08 m/s. The total error in three dimensions (3D) was computed for the position of the hand cluster from the target position trace over time. In each dimension, the target trajectory was a linear (constant velocity) position trace that directly (straight line) connects the initial hand position to a position near the sphere from which it can immediately be grasped. The time course of each target trajectory was coincident with the 4 s duration of the constant-speed pacers. Additional performance metrics evaluated in this study involved efficiency of movement. These metrics included greater smoothness (Hogan and Sternad, 2009) and lower 3D acceleration given a constant velocity target. These movement performance metrics were explicitly computed for each trial as follows:

**Pathlength**(over entire reach) →

$$P=\sum_{i=1}^{N}\sqrt{{\left({\mathrm{px}}_{i+1}-{\mathrm{px}}_{i}\right)}^{2}+{\left({\mathrm{py}}_{i+1}-{\mathrm{py}}_{i}\right)}^{2}+{\left({\mathrm{pz}}_{i+1}-{\mathrm{pz}}_{i}\right)}^{2}}$$

where

*i* = time index

*N* = total number of time-points until grasp contact at sampling frequency (120 Hz)

*px*, *py*, *pz* = *x*, *y*, *z* position of hand marker-cluster

$$\mathrm{Inverse Pathlength}\to P^{-1}=\frac{1}{P}$$

Kinematics (ateachtimeindex)→

$$vx_{i+1}=\frac{px_{i+1}-px_{i}}{\Delta t},ax_{i+1}=\frac{vx_{i+1}-vx_{i}}{\Delta t},jx_{i+1}=\frac{ax_{i+1}-ax_{i}}{\Delta t}$$

where

*vx*, *ax*, *jx* = velocity, acceleration, and jerk of hand marker-cluster in *x*-dimension (repeated for *y*- and *z*- dimension) at given time index. Δ*t* = 1/120 s. A moving mean window of 12 time points (0.1 s given sampling frequency of 120 Hz) was employed for smoothing kinematic trajectories.

Total 3D Acceleration(ateachtimeindex)→

$$Acc_{i}=\sqrt{ax_{i}^{2}+ay_{i}^{2}+az_{i}^{2}}$$

$$\mathrm{Total}\text{Smoothness}\left(\mathrm{over}\mathrm{entire}\mathrm{reach}\right)\to S_{tot}=Sx+Sy+Sz$$

where

$Sx=\sum_{i=1}^{N}jx_{i+1}^{2}$ (smoothness in each dimension, e.g., *x*-dimension)

$Sx^{\prime }=Sx\frac{D^{3}}{vx^{2}}$ (unitless smoothness in each dimension)

*D* = total duration of reach

*vx* = mean velocity in *x*-dimension during reach

$$\mathrm{Inverse Smoothness}\to S_{tot}^{-1}=\frac{1}{S_{tot}}$$

$$\mathrm{Controller Efficiency}\to CE=\frac{S_{tot}^{-1}}{Acc}$$

where

*Acc* = mean total 3D acceleration during reach

The following statistical analyses were performed:

• For comparisons across tested control modes, a Kolmogrov–Smirnov confirmed normality in analyzed data sets and use of parametric statistical tests. Repeated-measures one-way ANOVA was done independently on data for agency and each reach-to-grasp performance metric across the single factor of control modes. *Post hoc* comparisons between paired test cases were made with Bonferroni correction for multiple comparisons. The *p*-value, F-statistic, and eta-squared metric were reported for significance and effect size.
• In assessing dependence of a performance metric on agency, a linear regression analysis was applied to identify evident relationships of performance or explicit agency to implicit agency. The F-statistic and *p*-value was computed to refute the null hypothesis that the slope coefficient was equal to zero and suggest significant dependence on implicit agency. A significant non-zero slope indicated a simple relationship between either a performance metric or explicit agency to implicit agency. The actual slope value indicated the magnitude of dependence of each variable on implicit agency.
• An unpaired *t*-test (two-tailed) was used to assess possible significant difference in agency between the grasp-only and the five test cases for reach-to-grasp.

A paired *t*-test (two-tailed) was used to assess difference in agency and performance between the Baseline test block at the start of the session versus the end of the session.

## Results

This study demonstrates the effects of varying control modes of a virtual hand on agency and performance of reach-to-grasp. Results are organized as follows: preliminary considerations of agency and performance of the reach-to-grasp task, agency and performance across control modes, changes in movement efficiency (e.g., smoothness) across control modes, and path length kinematics during high agency versus low agency.

### Preliminary Considerations of Reach-to-Grasp Agency and Performance

**FIGURE 4:**
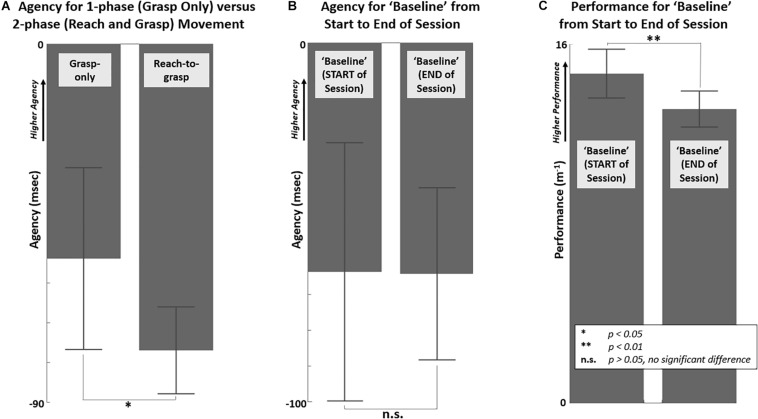
Differences in agency and performance shown between “grasp only” and “reach and grasp” and across the session. **(A)** Agency for “grasp only” versus all reach-to-grasp test blocks (*p* = 0.017, *t*-stat = 2.92), **(B)** Agency for “Baseline” test blocks at start versus end of session (*p* = 0.96, *t*-stat = 0.05), **(C)** Performance, measured as inverse of mean path length error, for “Baseline” test blocks at start versus end of session (*p* = 0.002, *t*-stat = 4.36).

The reach phase decreased agency of grasp compared to the grasp-only test case as shown in Figure 4A. No significant change in agency was observed for between the Baseline test blocks across the session (Figure 4B). There was a significant reduction in reaching performance (inverse of mean error to minimal path length trajectory) between the Baseline test block from start (14.7 m^–1^) to end (13.1 m^–1^) of the session (Figure 4C). Due to the observed reduction in Baseline performance, performance data across the session were adjusted by a linear correction factor. The correction factor was applied uniformly across sequential test blocks proportional to the reduction in Baseline performance from start to end of the session.

### Effect of Control Mode on Agency and Reaching Performance

**FIGURE 5:**
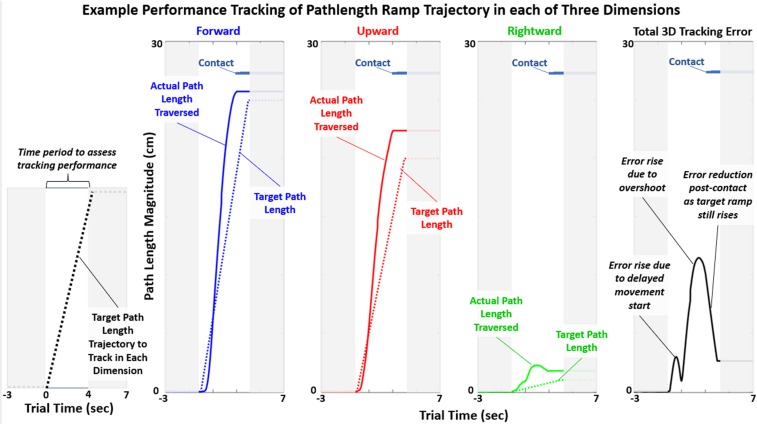
Example tracking of target path length shown for one subject during “Baseline” test case. The target path length changes linearly in time (“ramp”) in each of the three dimensions (3D). The primary performance metric in this study was the average total 3D tracking error during the time period of the target ramp (between *t* = 0 and 4 s).

The mean total 3D tracking error of the target minimal pathlength across time was the primary performance metric in this study. Example performance to track a minimal path length trajectory is shown in Figure 5. There was typically a delay in movement initiation despite a preparatory countdown cue. There was also tendency to move the virtual hand faster than the target constant velocity. This resulted in a quick overshoot of the target and completion of contact prior to completion of the target ramp trajectory.

**FIGURE 6:**
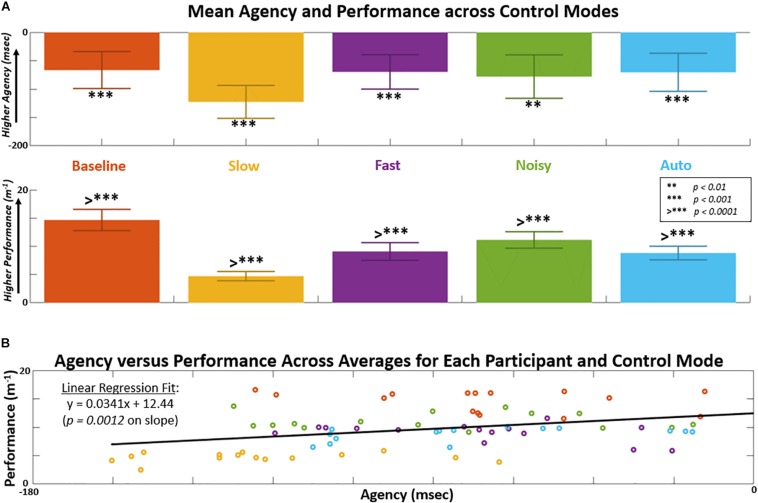
Control mode effects shown for agency and performance. Lowest pairwise *p*-value < alpha level indicated for each control mode from *post hoc* comparisons. **(A)** Agency positively measured according to underestimation of time-interval between grasp action and proceeding sound event. Performance positively measured as inverse of 3-D position error of reaching hand from target minimal path length trajectory. **(B)** Linear regression applied on data points of mean performance and mean agency across respective subject and control mode. Slope parameter from linear regression indicates a significant (non-zero, *p* < 0.01) positive relationship between agency and performance. F-stat for regression is 11.43 with *p* = 0.0012.

One-way ANOVA indicated significant differences in both agency (*p* < 0.001) and performance (*p* < 0.0001) across the single factor of control modes (Figure 6A and Table 1). The highest mean value in agency and performance was observed for the Baseline control mode. The lowest mean value in agency and performance was observed for the Slow control mode. The F-stat for both agency and performance were notably greater than 1 and with notable effect size (η^2^ > 0.30). A linear regression was applied to subject-averaged sample points for agency versus performance across all control modes tested (Figure 6B). The slope parameter was significantly greater than zero (*p* < 0.01) indicating a positive relationship between agency and performance.

**TABLE 1A:** Mean value comparisons for implicit agency and performance of minimizing reach pathlength across control modes.

	Control mode					ANOVA		
Metric	Baseline	Slow	Fast	Noisy	Auto	F-Stat	*p*-val	η ^2^
Implicit agency (ms)	−6632	−12229	−6930	−7738	−7033	7.58	3.94E-05	0.30
Performance (m^–1^)	14.71.8	4.70.8	9.11.6	11.11.5	8.81.2	96.6	9.81E-28	0.85

**TABLE 1B:** *Post hoc* comparisons (*p*-values) between control modes for implicit agency.

	Control mode				
Control mode	Baseline	Slow	Fast	Noisy	Auto
Baseline	–	**1E-04**	0.99	0.87	0.99
Slow	–	–	**4E-04**	**4E-03**	**4E-04**
Fast	–	–	–	0.96	0.99
Noisy	–	–	–	–	0.97

**TABLE 1C:** *Post hoc* comparisons (*p*-values) between control modes for performance (minimizing reach pathlength).

	Control mode				
Control mode	Baseline	Slow	Fast	Noisy	Auto
Baseline	–	**1E-08**	**1E-08**	**4E-08**	**1E-08**
Slow	–	–	**1E-08**	**1E-08**	**1E-08**
Fast	–	–	–	**1E-08**	0.98
Noisy	–	–	–	–	**3E-04**

*All post hoc comparisons made with Bonferroni correction. Significant post hoc p-values (<0.05) bolded.*

**FIGURE 7:**
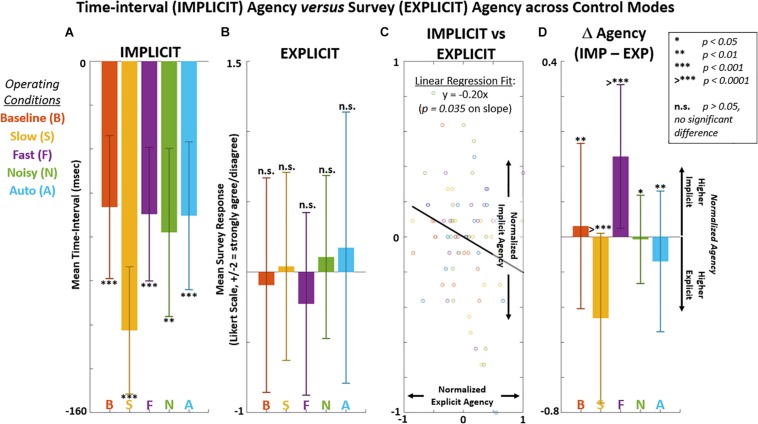
Comparing mean agency from IMPLICIT time-interval estimates (in ms) versus EXPLICIT survey responses (average Likert score) for each control mode. **(A)** Positive implicit agency is indicated as underestimation of actual time-intervals. **(B)** Positive explicit agency is indicated by level of agreement that the displayed control of the virtual hand reflects participant intent. Survey Likert scores given as: -*2 = Strongly Disagree*,−*1, Disagree, 0 = Neutral, 1 = Agree, 2 = Strongly Agree.* **(C)** Implicit versus explicit agency across subjects and control modes after self-normalizing for mean to equal zero and range over [–1, 1]. F-stat for regression is 4.62 with *p* = 0.035. **(D)** Relative shift shift from explicit to implicit shown for each control mode.

Implicit measures of agency using intentional binding are shown against survey-based explicit measures of agency in Figure 7 and Table 2. Significant differences (*p* < 0.05) in explicit agency were not observed across control modes (Figure 7B). Implicit and explicit agency results across subject-mode pairs were self-normalized [mean = 0, range over (−1, 1)] and plotted against each other in Figure 7C to suggest an inverse relationship (linear regression slope < 0, *p* < 0.05) in this study. The average difference in normalized explicit agency from implicit agency for each control mode is shown in Figure 7D. Across control modes, the normalized differences between explicit and implicit agency produced notable F-stat (9.88) and effect size (η^2^ = 0.36). The largest differences were observed for the Slow and Fast mode with a shift toward explicit and implicit agency, respectively.

**TABLE 2A:** Mean value comparisons for implicit and explicit agency across control modes.

	Control mode					ANOVA		
Metricm	Baseline	Slow	Fast	Noisy	Auto	F-Stat	p-val	η ^2^
*Explicit Agency (Likert)*	−0.090.76	0.040.67	−0.230.65	−0.110.58	0.170.96	0.7113	0.59	0.04
*Normalized Δ Agency (Implicit – Explicit)*	0.030.24	−0.230.24	0.230.20	−0.0070.13	−0.070.20	9.88	2.2E-06	0.36

**TABLE 2B:** *Post hoc* comparisons (*p*-value) between control modes for difference (shift) in normalized agency, Δagency = implicit – explicit.

	Control mode				
Control mode	Baseline	Slow	Fast	Noisy	Auto
*Baseline*	–	**7E-03**	7E-02	0.99	0.67
*Slow*	–	–	**5E-07**	**3E-02**	0.21
*Fast*	–	–	–	**2E-02**	**2E-03**
*Noisy*	–	–	–	–	0.92

*All post hoc comparisons made with Bonferonni correction. Significant post hoc p-values (<0.05) bolded.*

### Effect of Control Mode on Movement Efficiency

**FIGURE 8:**
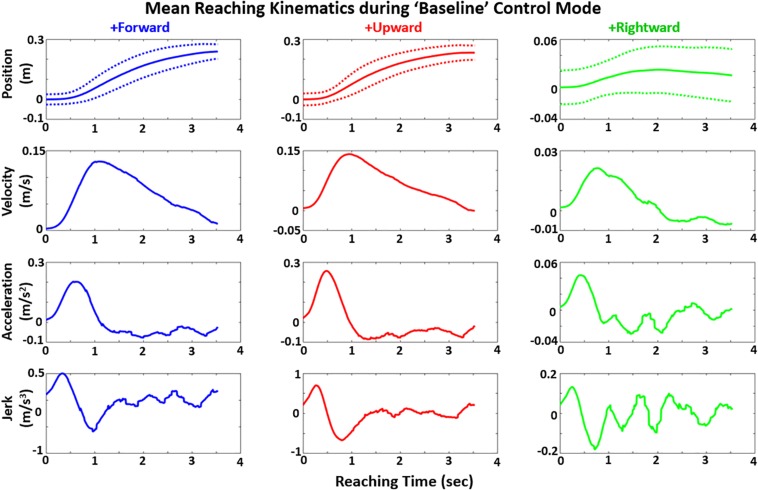
Mean position (with standard deviation dotted bands), velocity, acceleration, and jerk profiles for reaching hand shown in each movement direction for “Baseline” control mode across all subjects. For these mean trajectory plots, the trajectory of each subject was projected to fit across the average reach-to-contact time of 3.49 s for all subjects prior to trajectory averaging.

The mean kinematic trajectory for reach in each direction is shown for Baseline in Figure 8. Given the reach-to-grasp task is continuous with clear initiation and termination, the movement smoothness was computed based on minimization of integrated squared-jerk (Flash and Hogan, 1985) for each control mode. To remove dependencies on movement duration or amplitude, the squared-jerk term is made unitless (Hogan and Sternad, 2009) based on movement time and mean velocity in each direction.

**FIGURE 9:**
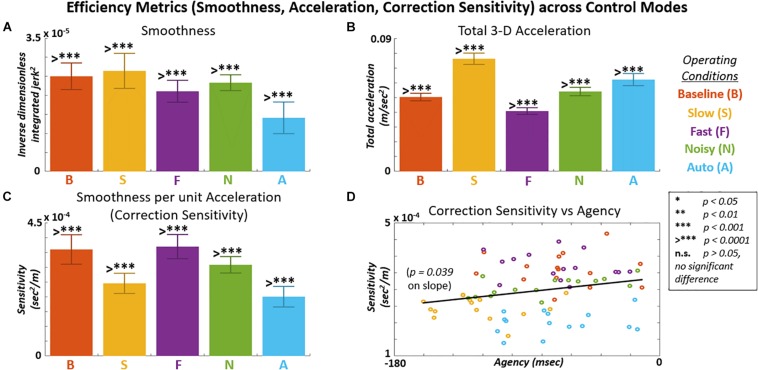
Various metrics of movement efficiency shown across control modes. **(A)** Smoothness values made dimensionless and inverted so higher values indicate greater smoothness. **(B)** Total acceleration in 3-D indicates magnitude of corrections made in tracking constant velocity target trajectory. **(C)** Smoothness per acceleration (correction sensitivity) computed to indicate smoothness achieved as a function of correction effort. **(D)** Correction sensitivity positively related to implicit agency across subjects and control modes (F-stat = 4.40, *p* = 0.039).

Results for select metrics of movement efficiency across control modes are shown in Figure 9 and Table 3. Smoothness (Figure 9A) is shown as the inverse of the integrated unitless squared-jerk metric summed in all three directions. The inverse operation presents higher smoothness by higher positive value. Highest smoothness was observed for the Slow control mode. However, the highest total 3-D acceleration (Figure 9B) was also observed for the Slow control mode. Higher acceleration indicates greater corrections were made online in tracking a constant-velocity movement target. When smoothness is normalized by total 3-D acceleration (Figure 9C), then the highest smoothness per unit acceleration was achieved during the Baseline and Fast control modes. Higher smoothness per unit acceleration suggests greater sensitivity of efficiency to a given correction, i.e., “correction sensitivity.” Correction sensitivity is plotted against agency for data points across subjects and control modes in Figure 9D. A linear regression on that data indicates a positive relationship (slope > 0, *p* < 0.05) between correction sensitivity and agency.

**TABLE 3A:** Mean value comparisons for performance efficiency metrics across control modes.

	Control mode					ANOVA		
Metric	Baseline	Slow	Fast	Noisy	Auto	F-Stat	p-val	η ^2^
*Smoothness (unitless, 10*^–^*^5^)*	2.50.34	2.60.46	2.10.29	2.30.21	1.40.41	27.8	7.6E-14	0.61
*Total Acceleration (cm/sec^2^)*	5.00.2	7.60.4	4.10.2	5.40.3	6.20.4	260.3	3.2E-41	0.94
*Correction Sensitivity (sec^2^/m, 10*^–^*^4^)*	3.60.5	2.50.3	3.70.4	3.10.3	2.00.3	55.0	6.7E-21	0.76

**TABLE 3B:** *Post hoc* comparisons (*p*-value) between control modes for smoothness.

	Control mode				
Control mode	Baseline	Slow	Fast	Noisy	Auto
*Baseline*	–	0.81	**2.7E-02**	0.69	**9.9E-09**
*Slow*	–	–	**9.3E-04**	0.12	**9.9E-09**
*Fast*	–	–	–	0.42	**9.2E-06**
*Noisy*	–	–	–	–	**1.8E-08**

**TABLE 3C:** *Post hoc* comparisons (*p*-value) between control modes for total acceleration.

	Control mode				
Control mode	Baseline	Slow	Fast	Noisy	Auto
*Baseline*	–	**9.9E-09**	**1.0E-08**	**1.7E-02**	**9.9E-09**
*Slow*	–	–	**9.9E-09**	**9.9E-09**	**9.9E-09**
*Fast*	–	–	–	**9.9E-09**	**9.9E-09**
*Noisy*	–	–	–	–	**2.8E-08**

**TABLE 3D:** *Post hoc* comparisons (*p*-value) between control modes for correction efficiency (smoothness over acceleration).

	Control mode				
Control mode	Baseline	Slow	Fast	Noisy	Auto
*Baseline*	–	**9.9E-09**	0.96	**3.5E-03**	**9.9E-09**
*Slow*	–	–	**9.9E-09**	**2.5E-04**	**1.5E-02**
*Fast*	–	–	–	**3.4E-04**	**9.9E-09**
*Noisy*	–	–	–	–	**1.0E-08**

*All post hoc comparisons made with Bonferonni correction. Significant post hoc p-values (<0.05) bolded.*

### Effect of High Versus Low Agency on Path Length Kinematics

**FIGURE 10:**
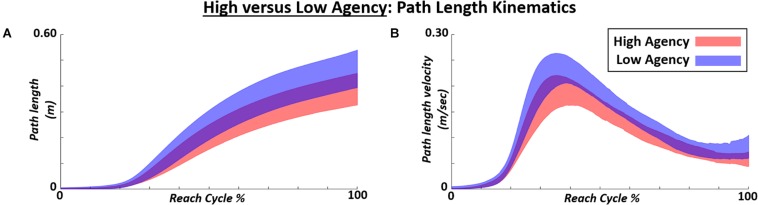
Mean path length kinematics for position (m) and velocity (m/sec) shown for high implicit agency (top 50%) trials across all subjects and control modes versus low implicit agency (bottom 50%) trials. Kinematics presented as path length position **(A)** and velocity **(B)** across reach cycle%. Path length position and velocity plotted as mean +/–1 standard deviation varying across reach cycle for all participants tested.

The general effects of high versus low implicit agency on path length position and velocity over the reach cycle are shown in Figure 10. The mean path kinematic trajectories are shown across the top (high) 50% of trials in agency versus the bottom (low) 50% of trials across all participants and control modes. High agency trials generally demonstrate shorter path length trajectories and slower path length velocities throughout the reach cycle.

**FIGURE 11:**
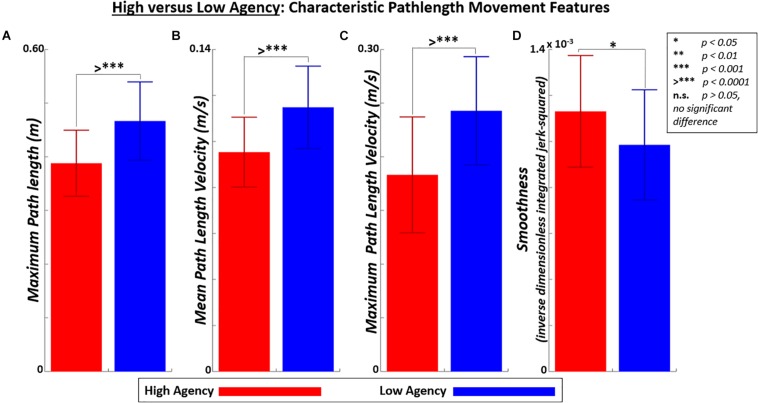
Characteristic pathlength movement features shown for high implicit agency (top 50%) trials versus low implicit agency (bottom 50%) trials. Movements features include: **(A)** maximum path length (*p* = 1E-05, *t*-stat = 5.10), **(B)** mean path length (*p* = 9E-06, *t*-stat = 5.13), **(C)** maximum path length velocity (*p* = 5E-05, *t*-stat = 4.57), and **(D)** smoothness (*p* = 0.012, *t*-stat = 2.62).

Figure 11 indicates that high agency trials produce significant (*p* < 0.001) reductions in the following movement features of path length: maximum path length, mean path length velocity, and maximum path length velocity. These high agency effects were desirable given the performance task was to minimize path length, ideally by following a minimum path length trajectory of 0.32 m at a constant velocity of 0.08 m/s. Figure 11 also indicates a significant increase (*p* < 0.05) in movement smoothness in path length with high agency.

## Discussion

This study demonstrated a positive relationship between agency of grasp and performance of reach-to-grasp across various control modes of the virtual hand. Implicit agency was measured through intentional binding of grasp action, and performance was primarily assessed as inverse of mean reaching error to a minimized path length trajectory. The results of this study may establish motivation for adapting user-device interfaces to co-maximize agency and performance. Of special interest are devices for movement assistance and rehabilitation, such as prostheses and exoskeletons. Clinical paradigms for motor rehabilitation that offer high value in both user engagement and functional utility have the best chances for retention and success (Wulf et al., 2010). To this end, the flexibility and accessibility of VR environments can be well leveraged to adapt rehabilitation platforms that co-maximize agency and movement performance. While implicating greater user agency over a device with higher functional performance is intuitive, the agency-performance link for movement has not been clearly established previously. This study relates agency, the autonomous sense of control, to functional performance across several control modes that can be standardly adapted for rehabilitation devices or rehabilitation training paradigms.

This study demonstrated a significant positive relationship (*p* < 0.001, Figure 6) between grasp agency and reaching performance across five distinct modes of control. This study also indicated how agency of grasp action is reduced in the presence of a preceding reach (Figure 4) compared to agency of movement initiation (Haggard et al., 2002). This is an important result since it suggests how agency of complex task action is modulated due to intermediate movement stages, which are further modified in this study with each control mode. The tested control modes were chosen to reflect control features (speed, noise mitigation, automaticity) commonly tuned for a movement device. The overall positive relationship between performance and agency is driven by the relatively high-agency, high-performing “Baseline” case and the relatively low-agency, low-performing “Slow” case. The “Fast” case yielded moderate-agency and moderate-performance. In total, these observations suggest that control sensitivity of speed may be a key tuning parameter for a device to co-maximize both user agency and functional performance.

Given high agency and performance for “Baseline,” it may be especially important to tune motion of a device to best match that of intact or restored proprioception and kinesthesia (Marasco et al., 2018). “Baseline” may also best facilitate the positive contributions of embodiment onto agency (Caspar et al., 2015). The “Slow” condition demonstrated the lowest agency, which indicates that experiencing slower device speeds relative to one’s intent significantly reduced the sense of control. For “Slow,” participants were required (unintentional) to move their own hands faster to compensate for the visual lags they observed for the virtual hand. While greater *intentional* effort can produce greater agency (Minohara et al., 2016), greater unintentional effort may reduce perceived efficacy of user control, especially if it promotes feelings of inability to initiate faster speeds (Kawabe, 2013). In this study, slower and faster speed control of the virtual hand required participants to actually reach longer and shorter, respectively. This limitation was required to employ changes in control speed while ensuring the task pathlength of the virtual hand was constant.

The remaining control mode cases were “Noisy” and “Auto,” which were categorically different from the other three which can be related by speed. These cases generally produced intermediate agency and performance relative to “Baseline” and “Slow.” “Noisy” may have been cognitively distracting in this study, but previous work has suggested that sensory noise (Collins et al., 2003) can improve motor function or indicate natural tremor (Allum, 1984; Riviere and Thakor, 1996) to better reflect human motion. For noise to be a cognitive or performance enhancer, there may be additional considerations beyond the scope of this study such as identifying a custom resonant frequency for each person. “Auto” would expectantly reduce agency given its intended feature to remove control from the user. It has been shown that increased automation can reduce sense of agency during aircraft control (Berberian et al., 2012), and that intentional binding is sensitive to degrees of automaticity. Our study similarly uses intentional binding to indicate a reduction in agency with increased automation of a movement device. Bang-bang (abrupt switch between on-off states) control is an underlying principle in automating natural movement (Ben-Itzhak and Karniel, 2008) and powered movement assistance (Farris et al., 2013). To facilitate greater user agency over a rehabilitation device, the level of proportional control (Lenzi et al., 2012) must be optimized.

While agency is a measure of subjective perception, its implicit quantification through intentional binding and positive relationship to performance suggests its plausible incorporation in engineering better movement systems. For comparison to explicit agency (Moore et al., 2012), participants provided Likert-scale survey responses, but only after each trial block, as in Berberian et al. (2012). Since performance and implicit measures were taken after each trial, no conclusions between explicit agency and performance were made in this study. While implicit and explicit measures of agency may expectedly be related, they indicate agency at different levels. With implicit agency there is low-level and non-conceptual formation of being an agent, while explicit attribution of agency involves higher-order judgment (Moore et al., 2012). There has been compelling suggestion that there are separable implicit and explicit learning systems in dissociating their effects. Perruchet et al. (2006) demonstrated how, for a probabilistic learning task pairing two events, greater prediction strength was observed with implicit learning which relied more on recency effect. In our study, there appeared to be an inverse relationship between implicit and explicit measures of agency (Figure 7C), indicating separate levels of perceived learning. There also appears to be larger shifts toward implicit agency with the “Fast” case but toward explicit agency with “Slow.” This result suggests that perceptions of probabilistic learning and conscious judgment are sensitive to speed in this study and should be considered accordingly for potential device adaptation.

We next investigated metrics for movement control efficiency (increased smoothness, decreased acceleration, change in smoothness per change in acceleration) across control modes and their dependence on agency. Since agency is affected by perception of outcome and effort, efficiency of a movement device is implicated with agency and should be considered in optimizing user-device integration. Against the hypothesis that agency produces better movement characteristics, the “Slow” case, which demonstrated the lowest agency, also exhibited the highest smoothness (Figure 9A). Further inspection showed how this smoothness came at a cost of higher corrective accelerations to modulate biomechanical control (Winter, 2009), otherwise minimized for a constant velocity task. When smoothness is normalized by total accelerations, a metric for correction sensitivity was inferred. For “Slow,” this sensitivity is significantly lower than “Baseline” or “Fast,” just as with agency and tracking performance. Across all five control modes, there is an apparent positive relationship (*p* < 0.05 on linear regression slope) between correction sensitivity and agency (Figure 9D). Participants were not aware of considering any efficiency metrics, but control modes producing higher agency may produce performance benefits at multiple levels (execution and efficiency).

Finally, the effects of generally high (top 50%) agency were also observed on general path length kinematics (Figure 10) and specific pathlength characteristics (Figure 11). High agency generated reduced path length (primary performance task objective), reduced mean and peak path length velocity (closer to target constant velocity of 0.08 m/s^2^), and smoother (more efficient) movement. While this study primarily aimed to demonstrate performance and agency modulation across control modes as motivation for device adaptation, a general positive relationship between agency and movement performance was also apparent.

## Conclusion

In conclusion, this study has demonstrated clear dependence between implicit agency, based on time-interval estimation, and reaching performance across varying control modes. This dependence is apparent across conditions of speed changes, inclusion of noise, and adding a measure of automation. This suggests the potential for adapting control of devices, such as those for movement assistance, to co-maximize cognitive agency and performance. While performance indicates greater functional abilities, higher agency facilitates cognitive integration between user and device for ease-of-use and more natural control. Agency may also be key in accelerating learning and clinical retention of rehabilitation devices. Implicit measures of agency based on intentional binding are potentially reliable foundations for observing positive agency-performance dependencies.

This study was conducted in VR to ensure the pathlength for the reach-to-grasp task was visually similar while systematically varying control modes. It remains unclear how VR is best employed to identify optimal control modes for real-world devices. The objective with this work was to demonstrate the positive relationship between implicit agency and performance and their dependencies across control modes. This finding should then inspire practical methods that robustly and automatically adapt device control for each user toward greater agency and performance. Virtual reality could be a highly efficient medium in which to identify initial user-fitted control parameters. Those parameters may then be further refined based on real-world observations. Similar approaches have been utilized whereby computational models indicate basic operating characteristics of a control system (Nataraj et al., 2010, 2012c) prior to implementation in a clinical setting (Nataraj et al., 2012a, b).

In the future, alternative measures of agency such as neurophysiological recordings may be more robust for control system adaptation. Characterizing agency according to patterns in muscle electromyography (EMG) or brain electroencephalography (EEG) would be practically beneficial. These recordings often serve as command inputs to control systems for movement devices. Furthermore, neurophysiological recordings would not necessitate conscious user responses during adaptation of device control. Reducing such user onus could mitigate cognitive fatigue although that was not readily apparent in this study. Meanwhile, implicit agency through time-interval estimates could be critical in identifying what neurophysiological patterns best represent cognitive states of high agency. Changes in EEG readiness potential have been shown with greater agency in relation to the intent to initially move (Jo et al., 2014). However, spectral coherence changes in EEG and EMG during high agency movement remains unclear. Ultimately, clear biomarkers for high agency and performance would be invaluable in optimizing user-device interfaces for movement through better musculoskeletal control systems (Nataraj et al., 2010, 2012b).

## Data Availability Statement

The datasets generated for this study are available on request to the corresponding author.

## Ethics Statement

The studies involving human participants were reviewed and approved by the Stevens Institute of Technology IRB. The patients/participants provided their written informed consent to participate in this study. Written informed consent was obtained from the individual(s) for the publication of any potentially identifiable images or data included in this article.

## Author Contributions

RN: designing and developing the experiment, analyzing the data, writing and revising the manuscript, and directing the project. SS: recruiting participants, performing the data collections, and revising the manuscript. AS: recruiting participants and performing the data collections. ML: revising the manuscript.

## Conflict of Interest

The authors declare that the research was conducted in the absence of any commercial or financial relationships that could be construed as a potential conflict of interest.
